# Differential diagnosis problems in a patient with dysphonia and chronic lymphocytic leukemia

**DOI:** 10.12669/pjms.311.6091

**Published:** 2015

**Authors:** Gabriela-Ariadna Gavrila, Romeo-Gabriel Mihaila, Ioan Manitiu

**Affiliations:** 1 Gabriela-Ariadna Gavrila, MD, Emergency County Clinical Hospital Sibiu, Corneliu Coposu Avenue, No 2-4, 550245, Sibiu, Romania.; 2 Romeo-Gabriel Mihaila, MD, PhD, “Lucian Blaga” University of Sibiu, Faculty of Medicine, Str. Lucian Blaga, nr. 2A, Cod 550169, Sibiu, Romania.; 3 Ioan Manitiu, MD, PhD, “Lucian Blaga” University of Sibiu, Faculty of Medicine, Str. Lucian Blaga, nr. 2A, Cod 550169, Sibiu, Romania.

**Keywords:** Chronic lymphocytic leukemia, Dysphonia, Lung cancer, Mediastinal compression, Genomic instability

## Abstract

Dysphonia is frequently an expression of laryngitis, especially when it comes in the evolution of an immunosuppressed patient, as happens in chronic lymphoproliferation. But other causes of dysphonia should also not be forgotten, including the possibility of new malignancies, especially due to the fact that these patients have genomic instability that predisposes to appearance of a second or even a third cancer. We present the case of a patient who developed dysphonia during chronic lymphocytic leukemia evolution. Its etiology was a mediastinal compression through lymph nodes, not linked to leukemia, but produced by metastases of a bronchopulmonary cancer, appeared recently. Dysphonia condition due to vocal cord dysfunction must include diseases of the mediastinum, the neck and the brain stem. The rapid and correct diagnosis and the prompt start of an appropriate treatment are of paramount importance for clinician who manage their care and for patient survival.

## INTRODUCTION

Dysphonia is defined as voice disorders like speech impairment or difficulty (hoarseness, weakness or even loss of voice), often due to a physical disorder of the vocal cord. Sound is produced by vibration of the vocal cords epithelium and therefore any factor that modifies air passage through the larynx (scars, weakness, spasms of vocal cords) alters the sound-production mechanism.^1^

Among the most common causes of dysphonia reported to ENT (ear, nose and throat) specialists are:

- Infectious condition like acute or chronic laryngitis is the most common cause of dysphonia.
- Cysts, papillomas, polyps or lumps on vocal cords usually result from vocal abuse to singers (e.g. singer’s nodules), sport commentators, teachers, impede with their normal closing during speaking.
- A pretty common cause of dysphonia, particularly in the morning is gastroesophageal reflux of acid from the stomach up to larynx.
- Thyroid conditions - most notably untreated hypothyroidism and acromegaly.
- Larynx trauma - intubation during surgery, trauma during a bronchoscopy, major trauma to the throat region during traffic accidents, surgery to the vocal cords, will cause scarring and dysfunction of the voice.
- Any surgery (thyroid, heart or head and neck surgeries) in the region where a nerve travels can induce laryngeal nerve paralysis, temporary or permanent.
- Local larynx irritants - inhaled corticosteroids long-term use for chronic obstructive pulmonary disease, asthma.
- Reinke’s oedema of the larynx.
- Psychological conditions - voice changes as a reaction to day-to-day stress.
- Neurological conditions like myasthenia gravis, multiple sclerosis, strokes, Parkinson’s disease due to their effects on the nerves serving the vocal cords.
- Cancer - sometimes dysphonia is the first symptom in the neoplasm of the larynx, the pharynx (the throat), the lungs, the thyroid and lymphoma. Mediastinal metastases from the breast, lungs or other cancers of the body can press on nerves directing to the voice box.^2^^,^^3^

We not mentioned here rare or relatively uncommon diseases. We present an adult case developing dysphonia during chronic lymphocytic leukemia (CLL) evolution.

## CASE REPORT

A 60-year-old male patient was admitted to the hematology service with dysphonia, headache, asthenia, marked fatigue and generalized health deterioration. This patient with a past medical history of CLL Rai stage II, CD 38(+), ZAP 70(-), came to our attention during a certain period of hematological remission of CLL after performed 6 cycles of chemotherapy with fludarabine, cyclophosphamide, dexamethasone and rituximab.

The patient had a blood pressure of 110/70 mm Hg at presentation. The physical examination was abnormal with peripheral lymphadenopathy in both laterocervical and left axillary, spleen and liver enlargement being noted: the liver edge palpated 2 cm below the right costal margin, the spleen at 3 cm below left costal margin, confirmed by ultrasound. On auscultation we found vesicular murmur tightened. Laboratory work demonstrated leucocytosis (51.700 x 10^9^/mL) with a majority of small mature lymphocytes (67%), normal haemoglobin (13,2g/dL) and normal platelet count (219 x 10^9^/mL). The myelogramm shows a hypercellular marrow with marked diffuse small lymphocytic infiltration up to 90%. Immunophenotyping of peripheral lymphocytes confirmed the diagnosis of B chronic lymphocytic leukemia (B-CLL) with an expression of CD5, CD23, CD19. The lymphocytes expressed CD38 antigen, but ZAP70 was negative.

Blood chemistries were normal, and he had negative inflammatory tests. The chest computed tomography scan revealed two opacities located pretracheal and on the chest and a tissue mass on the mediastinum and on the left lung hil with loco-regional invasion, multiple bulky lymph nodes above and below diaphragm, and a liver mass of 1.7 cm. The skull computed tomography scan revealed: iodofile nodular masses located infra- and supratentorial (suggestive of brain metastases) and cortical atrophy. The bronchoscopy can evaluates the possible tumor extension and the obstructive phenomena. In this case, bronchoscopy showed normal trachea, but immobile left vocal cord. In addition, left lung lobe related bronchus was partially stenosed by extrinsic compression and lymphatic circulation disorders were present. Examination of bronchus brushing cytology showed no cellular atypia.

During hospitalization, the patient was depleted for signs of intracerebral hypertension and transferred to the oncology service due to the left lung cancer with brain metastases, where a palliative cerebral radiotherapy was recommended.

## DISCUSSION

Anatomically speaking the larynx and his intrinsic muscle are innervated by the left and right branches of the recurrent laryngeal nerve. These are leaving the vagus, passes *around* the right subclavian, respectively the arch of the aorta, cross the neck in the tracheoesophageal groove and enter the larynx. Therefore many types of lesions along the branches of cranial nerve X pathway, particularly the laryngeal recurrent nerves may result in paralysis of the vocal cord.^3^ Dysphonia condition due to vocal cord dysfunction must included diseases of the mediastinum, the neck and the brain stem. Any external compression by massive laterocervical lymphadenopathy occurred in the evolution of CLL in this area may affect the voice. On the other hand, the presence of tumor *metastases in the mediastinum because of *breast, lungs or other regions cancer can press on nerves leading to the larynx. Surgical injury or penetrating trauma to the areas between the lungs may also cause vocal cord paralysis.

About 10% of CLL patients will have their illness transformed into a Richter's syndrome (RS) after several years of evolution. This term refers to the development of an aggressive non-Hodgkin's lymphoma (diffuse large cell or immunoblastic) when during the course of CLL appear an abrupt clinical deterioration with worsening systemic symptoms, rapid tumor growth, and/or extranodal involvement. Accurate diagnosis involves tissue biopsy. This syndrome is characterized by highly resistant to current therapies and median survival approximately 6 months. Our patient with CLL, with evidence of disease progression (some small lymph nodes showed on CT scan 3 months ago, but without lung lesions) which comes to the hospital with malaise, hoarseness, fatigue, weight increase, leukcytosis with lymphocytosis and the appearance of new lymph nodes, enlarged liver and spleen raises the question of the disease progression. In addition, CLL CD38-positive is characterised by an unfavourable clinical course, rapid progress to advanced Rai stages, reduced responsiveness to fludarabine and shorter survival.^4^

Patients with CLL are predisposed to the development of a second malignancy due to impaired immune system, chemotherapy or genomic instability.^5^^,^^6^ CLL status is characterized by progressive impairment in both cell-mediated immunity (B-lymphocyte defects, abnormality T-cell number and function, natural killer defects) and humoral-mediated immunity (low gamma globulin levels).^7^ CLL therapeutic modalities, especially nucleoside analog therapy, make the immunosuppression deeper by involving all elements of the immune system and favoring infectious diseases. In the majority of cases, the second malignancy is developing after several years of CLL evolution and it is non hematological. Typically it is a period of spontaneous CLL remission who precedes by months or years the second malignancy occurrence (in as many as 33% of patients with CLL).^8^ It is well known the increased frequency (16%) of second malignancies in CLL and over two thirds of these patients will die this cause. Dysphonia is not a rare symptom in lung cancer who has a high prevalence. Dysphonia to lung cancer patients is according to underlying mechanisms: mediastinal disease, cervical lymphadenopathy, respiratory tract metastases, brainstem metastases, oropharyngeal infections, associated systemic disorders.

In both cases (lung cancer with brain metastases and RS) the life expectancy is often so short that the rapid and correct diagnosis and the promptly start of appropriate treatment are of great importance for clinician and for patient survival.
